# Association Between Endogenous Ketosis and Risk of Atrial Fibrillation in Intensive Care Versus General Ward Patients: A Retrospective Cohort Study

**DOI:** 10.3390/jcm15134966

**Published:** 2026-06-25

**Authors:** Kellina Maduray, Jingquan Zhong

**Affiliations:** State Key Laboratory for Innovation and Transformation of Luobing Theory, Key Laboratory of Cardiovascular Remodeling and Function Research of Chinese Ministry of Education, Chinese National Health Commission, Chinese Academy of Medical Sciences and Shandong Province, Department of Cardiology, Qilu Hospital of Shandong University, Jinan, Department of Cardiology, Qilu Hospital of Shandong University (Qingdao), Qingdao 266035, China

**Keywords:** atrial fibrillation, β-hydroxybutyrate, cardiac metabolism, intensive care unit, ketones, in-hospital, MIMIC-IV

## Abstract

**Background**: Metabolic reprogramming in critical illness and the physiological stress of general hospitalization represent fundamentally different states, yet it remains unknown if ketosis acts as a protective shield or a maladaptive metabolic response in the development of atrial fibrillation (AF) across these contexts. We examined urine and serum β-hydroxybutyrate measurements to understand the metabolic association among intensive care unit (ICU) and general hospital populations. **Methods**: This retrospective cohort study utilized the MIMIC-IV v3.1 database. Patients with preexisting AF or flutter were excluded. Ketosis was defined as urine ketone positivity (≥20 mg/dL) or serum β-hydroxybutyrate (≥1.0 mmol/L). The final analytic cohort included a general ward cohort (*n* = 13,641) and an ICU cohort (*n* = 10,251). Multivariable logistic regression, propensity score matching and subgroup analyses were performed. **Results**: In the ICU cohort, urine ketone positivity and elevated serum β-hydroxybutyrate were associated with lower incidence of AF (5.2% vs. 6.8%, *p* = 0.001; 3.1% vs. 9.4%, *p* = 0.034). After adjustment, urine ketone positivity remained independently associated with reduced odds of incident AF (adjusted OR 0.79, 95% CI 0.64–0.98, *p* = 0.032). Propensity-matched analyses demonstrated protective associations for urine ketones (OR 0.68, 95% CI 0.52–0.88, *p* = 0.004) and β-hydroxybutyrate (OR 0.24, 95% CI 0.08–0.70, *p* = 0.003). In contrast, urine ketone positivity in the general ward cohort was associated with higher incident AF (0.9% vs. 0.5%, *p* = 0.019) and increased adjusted odds (OR 2.62, 95% CI 1.03–6.66, *p* = 0.044). Urinary ketosis was associated with lower mortality and reduced inflammatory marker profiles across both the ICU and general ward cohorts. Subgroup analyses revealed directionally consistent ketone-AF associations across biological sex with no significant interaction effects. **Conclusions**: Endogenous ketones demonstrated a context-dependent association with incident AF across clinical acuity levels. These findings highlight ketone metabolism as a potential target for both arrhythmia monitoring and prevention.

## 1. Introduction

Atrial fibrillation (AF) is the most common arrhythmia encountered in clinical practice, affecting approximately 5% to 10% of patients in general hospital wards, while its incidence escalates significantly to 15% to 45% among critically ill patients in the intensive care unit (ICU) [1,2]. It is associated with increased risks of hemodynamic instability, thromboembolic events, prolonged hospitalization, and mortality [3,4,5]. In a stressed myocardium, the landscape is characterized by an energy deficit, where the heart’s traditional reliance on fatty acid oxidation becomes less efficient due to the high oxygen demand and mitochondrial inefficiency [6]. Ketone bodies, specifically serum β-hydroxybutyrate (β-OHB), have emerged as an oxygen-efficient alternative for adenosine triphosphate (ATP) production [7].

However, the role of ketones in cardiovascular health remains a subject of diverging hypotheses. Nielsen et al. and Yurista et al. have put forward the perspective that elevated ketones are a protective adaptive response that enhances myocardial resilience [8,9]. Conversely, Umpierrez and Korytkowski report that increased ketones in acute illness may simply be a maladaptive byproduct of metabolic failure [10]. This controversy is compounded as metabolic stress responses differ substantially between the ICU and general wards [11,12]. Such variations are consequential, as altered metabolism influences atrial electrophysiology through oxidative stress, mitochondrial redox balance, and ion channel stability [13,14].

The pathophysiological link connecting ketogenesis to atrial arrhythmogenesis suggests that ketone bodies may influence several pathways implicated in AF development. Increasing evidence indicates that metabolic remodeling plays a central role in the development of AF [15]. Mitochondrial dysfunction, oxidative stress, inflammation, and impaired myocardial energetics contribute to both atrial structural remodeling and electrophysiological instability [16,17,18]. Ketone bodies occupy a unique position within this framework, serving not only as alternative metabolic substrates but also as signaling molecules capable of influencing several pathways implicated in arrhythmogenesis [19].

In particular, β-hydroxybutyrate has been shown to modulate inflammatory signaling, reduce oxidative stress, and improve mitochondrial efficiency [20,21]. Experimental studies suggest that ketone metabolism may affect ion channel activity, myocardial substrate utilization, and electrophysiological homeostasis, potentially enhancing electrical stability during periods of metabolic stress [22,23]. Despite growing mechanistic evidence, the clinical relationship between endogenous ketosis and incident AF remains poorly understood, particularly across patient populations with differing levels of illness severity.

Ketone testing is not routinely integrated into standard clinical protocols and contributes a significant data gap in our understanding of how endogenous ketosis influences atrial arrhythmogenesis across different levels of illness acuity [24]. Moreover, the physiological divergence between circulating β-hydroxybutyrate and general ketonuria warrants careful consideration. Whereas ketonuria serves as a proxy for renal acetoacetate excretion, serum β-hydroxybutyrate functions as the metabolic driver of myocardial energetics [25,26]. Therefore, relying solely on urinary markers may mask the true relationship between circulating fuel availability and atrial stability [27,28].

In this study, we hypothesized that the metabolic impact of endogenous ketosis on atrial arrhythmogenesis is directed by patient acuity. The primary aim of this work is to understand the association between serum β-OHB, ketonuria, and incident AF across a diverse inpatient population. This investigation represents the first to quantify the effect of circulating β-hydroxybutyrate in the ICU setting. We further examined the direction and magnitude of this association using multivariable regression and propensity score–matched analyses between critically ill and non-critically ill populations.

## 2. Materials and Methods

### 2.1. Data Source

This study was conducted using data from the Medical Information Mart for Intensive Care IV (MIMIC-IV, version 3.1) database [29], a publicly available repository containing de-identified clinical information from patients admitted to the intensive care units of Beth Israel Deaconess Medical Center between 2008 and 2019. Access to the database was obtained after completion of the required Collaborative Institutional Training Initiative (CITI) certification (author credential ID: 15036091). In accordance with the Health Insurance Portability and Accountability Act (HIPAA) Safe Harbor provision, the dataset is fully de-identified; therefore, informed consent was not required for this study.

### 2.2. Study Population

**Figure 1:**
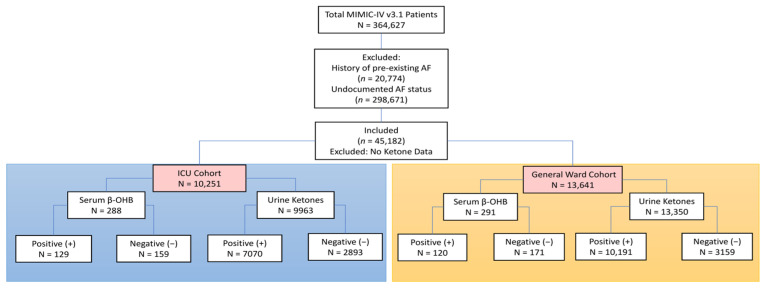
Study flowchart. The blue shaded area denotes the ICU cohort, and the orange shaded area denotes the General Ward cohort.

The initial screening identified 364,627 unique patients. Individuals with permanent or pre-existing AF or atrial flutter prior to admission were excluded. Patients without documented rhythm status during hospitalization were also excluded. Eligible participants were adults (age ≥18 years) who underwent ketone testing (urine ketones or serum β-hydroxybutyrate). Ketosis was defined as urine ketone positivity (≥20 mg/dL) or serum β-hydroxybutyrate (≥1.0 mmol/L) (Table 1). After applying these criteria, the final study population was two mutually exclusive cohorts based on level of care during hospitalization: a general ward cohort (*n* = 13,641) and an ICU cohort (*n* = 10,251). Patients were categorized according to urine ketone status and serum β-hydroxybutyrate levels recorded after admission and prior to the development of incident AF. Detailed cohort derivation and inclusion criteria are illustrated in Figure 1 in accordance with STROBE reporting guidelines.

### 2.3. Clinical Variables

All study variables were extracted using structured query language (SQL) in Google BigQuery (Google LLC, Mountain View, CA, USA; https://cloud.google.com/bigquery; accessed on 15 May 2026). For patients with multiple hospital admissions, only the index hospitalization (the first recorded admission within the database) for either the general ward or the ICU was included in the analysis to ensure independence of observations. Baseline ketone exposure was determined using the earliest available urine ketone or serum β-hydroxybutyrate measurement recorded after admission. To preserve temporal validity, only ketone measurements obtained prior to the occurrence of AF were included as exposure variables. The primary outcome of interest was the occurrence of incident AF during hospitalization. Baseline characteristics included demographic variables (age and biological sex), pre-existing comorbidities (hypertension, diabetes mellitus, heart failure, prior myocardial infarction, chronic obstructive pulmonary disease, stroke, obstructive sleep apnea syndrome, renal insufficiency, oncology, sepsis, and diabetic ketoacidosis), and medication exposure (insulin therapy, β-blocker use, and antiarrhythmic drug use). Clinical risk burden was evaluated using the Charlson Comorbidity Index (CCI) and CHA_2_DS_2_-VASc score. Vital signs included mean heart rate, mean arterial pressure, peripheral oxygen saturation (SpO_2_), and respiratory rate. Laboratory measurements included metabolic markers (glucose, lactate, uric acid, and phosphate), inflammatory indices (white blood cell count, neutrophil-to-lymphocyte ratio [NLR], red cell distribution width [RDW], and C-reactive protein), renal and organ-function markers (creatinine, blood urea nitrogen [BUN], BUN-to-creatinine ratio, lactate dehydrogenase, albumin, B-type natriuretic peptide, and troponin-T), electrolyte parameters (bicarbonate, potassium, and calcium), and hematologic variables (platelet count, hemoglobin, and hematocrit).

### 2.4. Statistical Analysis

Continuous variables are presented as median with interquartile range (IQR) to ensure consistency across study cohorts, whereas categorical variables are reported as frequencies and percentages. Cohort comparisons were performed using the independent samples *t*-test for normally distributed variables or the Mann–Whitney U test for non-normally distributed variables and the χ^2^ test or Fisher’s exact test for categorical variables, as appropriate. The association between ketone status and AF was evaluated using univariable and multivariable logistic regression analyses, with results reported as odds ratios (ORs) and 95% confidence intervals (CIs). Sequential adjustment models were constructed to assess the robustness of associations, including Model 1 (unadjusted), Model 2 (adjusted for age and biological sex), Model 3 (Model 2 plus comorbidities and medication exposure, including hypertension, diabetes mellitus, heart failure, prior myocardial infarction, stroke, chronic obstructive pulmonary disease, renal insufficiency, malignancy, insulin use, β-blocker use, and anti-arrhythmic medication use), and Model 4 (Model 3 plus adjustment for systemic inflammatory status using neutrophil-to-lymphocyte ratio [NLR]). High inflammatory burden was defined using a NLR dichotomized at the cohort median (5.85). To further reduce confounding, propensity score matching analyses and time-dependent Cox proportional hazards analysis were performed using the same covariates included in the multivariable regression models, and effects were estimated using corresponding odds ratios and hazard ratios (HRs), respectively. Subgroup analyses were conducted stratified by demographic and clinical characteristics, including age group, heart failure status, renal insufficiency status, and inflammatory burden, with interaction testing performed to evaluate potential effect modifications. All statistical tests were two-sided, and a *p*-value < 0.05 was considered statistically significant. Statistical analyses were performed using R (version 4.3.2; R Foundation for Statistical Computing, Vienna, Austria).

## 3. Results

### 3.1. Patient Demographics & Baseline Characteristics

A total of 10,251 ICU admissions and 13,641 general ward admissions were identified in the study cohort. Urine ketone measurements were available for 9963 ICU admissions and 13,350 general ward admissions and serum β-hydroxybutyrate measurements were available for 288 ICU patients and 291 general ward patients.

**Figure 2:**
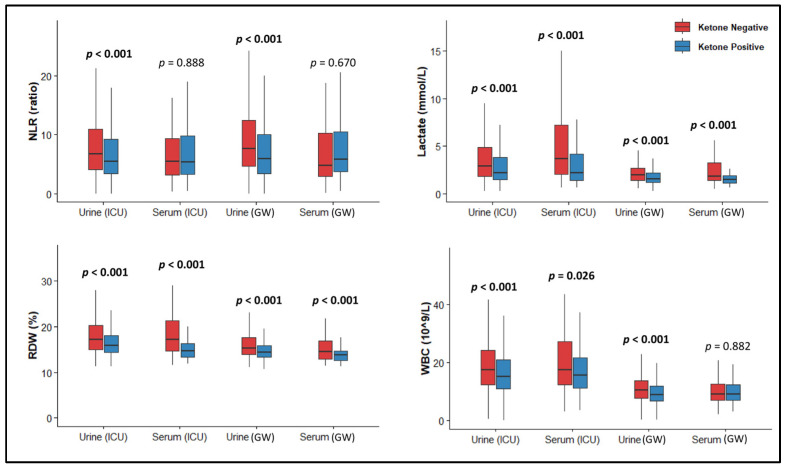
Boxplots comparing neutrophil-to-lymphocyte ratio (NLR), lactate, red blood cell distribution width (RDW), and white blood cell count (WBC) between ketone-positive and ketone-negative patients in the ICU and general ward (GW) cohorts, stratified according to urine ketone status and serum β-hydroxybutyrate levels.

Participants with positive urine ketones were older than those without ketonuria in both the ICU cohort (63 vs. 62 years, *p* = 0.014) and the general ward cohort (64 vs. 62 years, *p* < 0.001). Biological sex distribution was similar between ketone groups in the ICU cohort (*p* = 0.231), whereas a higher proportion of males was observed among ketone-positive patients in the general ward cohort (*p* < 0.001). Differences in comorbidities, metabolic indices, inflammatory markers, organ function parameters, and medication exposure between ketone groups are summarized in Table 2 and Table 3. Distributions of key inflammatory and metabolic biomarkers, including NLR, WBC, RDW, and lactate, are illustrated in Figure 2.

### 3.2. Incidence of AF and Mortality

Overall, de novo AF occurred in 582 patients (5.7%), and all-cause mortality occurred in 1814 patients (17.7%) in the ICU cohort. In the general ward cohort, de novo AF occurred in 113 patients (0.8%) and all-cause mortality occurred in 1243 patients (9.1%) (Table 4). The direction of association between urine ketone positivity and AF differed between ICU and general ward cohorts. In the ICU cohort, urine ketone positivity was associated with a significantly lower incidence of AF compared with urine ketone negativity (5.2% vs. 6.8%, *p* = 0.001). Similarly, higher serum β-OHB concentrations were associated with a lower incidence of AF (3.1% vs. 9.4%, *p* = 0.034). In contrast, within the general ward cohort, urine ketone positivity was associated with a higher incidence of AF (0.9% vs. 0.5%, *p* = 0.019), whereas no significant difference in AF incidence was observed between serum β-OHB groups (0.0% vs. 0.6%, *p* = 0.401). Mortality was significantly lower among urine ketone–positive patients in both ICU (13.9% vs. 27.3%, *p* < 0.001) and general ward cohorts (6.7% vs. 16.8%, *p* < 0.001). Similarly, higher serum β-OHB concentrations were associated with reduced mortality in both ICU (10.1% vs. 18.9%, *p* = 0.037) and general ward cohorts (5.0% vs. 13.5%, *p* = 0.018) (Table 5).

### 3.3. Multivariable Logistic Regression Analysis

In multivariable logistic regression models adjusting for demographics, comorbidities, medications, and inflammatory status, urine ketone positivity remained independently associated with reduced odds of AF in the ICU cohort (adjusted OR 0.79, 95% CI 0.64–0.98, *p* = 0.032). Elevated serum β-hydroxybutyrate demonstrated a trend toward reduced odds of AF in the ICU cohort (adjusted OR 0.25, 95% CI 0.06–1.02, *p* = 0.053). In contrast, urine ketone positivity in the general ward cohort was associated with increased odds of AF after adjustment (adjusted OR 2.62, 95% CI 1.03–6.66, *p* = 0.044). The direction and magnitude of associations remained consistent across sequential adjustment models (Table 6). In fully adjusted models, urine ketones remained independently associated with reduced mortality in both ICU (OR 0.44, 95% CI 0.39–0.50) and general ward cohorts (OR 0.41, 95% CI 0.35–0.47). The association for serum β-OHB was attenuated after adjustment (Table 7).

### 3.4. Propensity Score Matched Analysis

Propensity score matched analyses supported the protective association between ketone positivity and AF in the ICU cohort. After matching for demographic characteristics, comorbidities, medication exposure, and NLR, urine ketone positivity remained associated with reduced odds of AF (OR 0.68, 95% CI 0.52–0.88, *p* = 0.004). Similarly, elevated serum β-hydroxybutyrate concentrations were strongly associated with reduced AF incidence (OR 0.24, 95% CI 0.08–0.70, *p* = 0.003). The association between urine ketone positivity and AF in the general ward cohort was attenuated with a directional trend toward increased AF risk (Table 8). Findings were consistent for mortality, with urine ketone positivity associated with significantly lower mortality in both ICU and general ward cohorts, whereas associations for serum β-hydroxybutyrate were attenuated after adjustment (Table 9).

### 3.5. Subgroup Analyses

**Figure 3:**
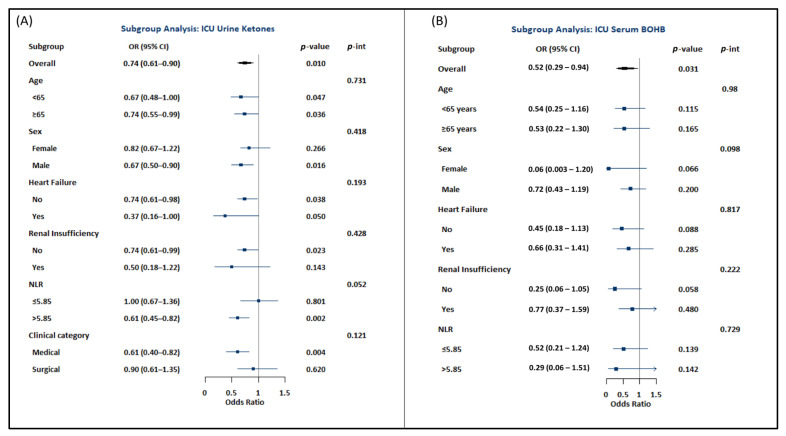
Forest plot of subgroup analyses evaluating the association between ketone status and incident AF in the ICU. Arrows indicate confidence intervals extending beyond the limits of the x-axis. (**A**) Urine ketone levels and incident AF in the ICU cohort. (**B**) Serum β-hydroxybutyrate and incident AF in the ICU cohort.

**Figure 4:**
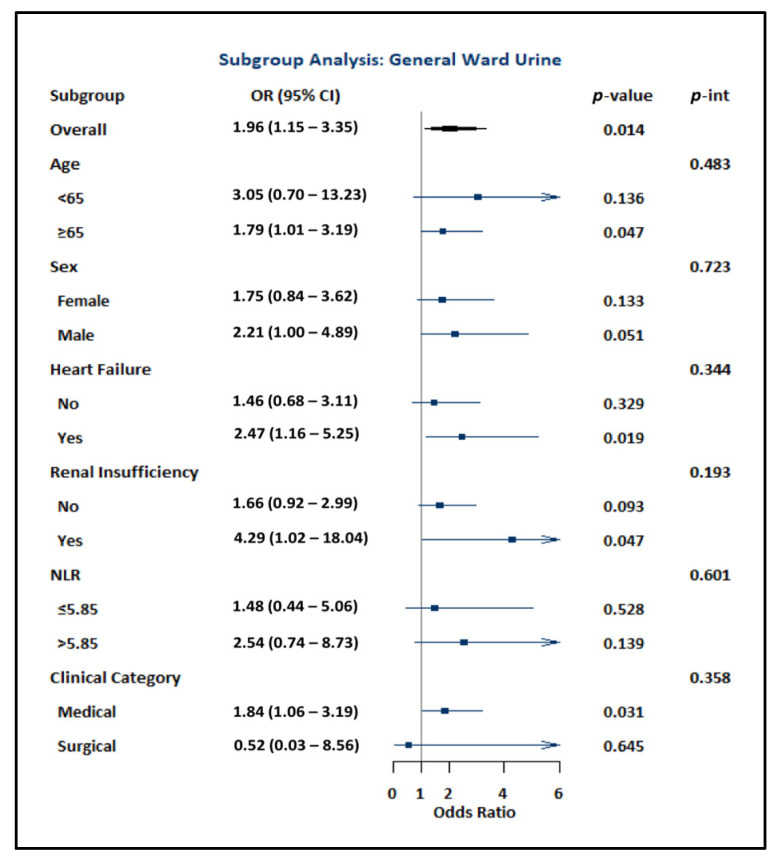
Forest plot of subgroup analyses evaluating the association between ketone status and incident AF in the general ward cohort. Arrows indicate confidence intervals extending beyond the limits of the x-axis.

Subgroup analyses demonstrated a consistent protective association between urine ketone levels and incident AF in the ICU cohort overall (OR 0.74, 95% CI 0.61–0.90, *p* = 0.01), with similar effects observed across age groups, male patients, individuals without heart failure or renal insufficiency, and those with elevated inflammatory burden (NLR > 5.85). No significant interaction effects were detected between urine ketone levels and subgroup variables. Serum β-OHB levels were likewise associated with reduced odds of AF overall (OR 0.52, 95% CI 0.29–0.94, *p* = 0.031). The general ward cohort analyses showed a positive association between urine ketone positivity and AF risk overall (OR 1.96, 95% CI 1.15–3.35, *p* = 0.014), with statistically significant associations observed in older patients and in those with heart failure or renal insufficiency. However, no significant interaction effects were identified across subgroup strata (Figure 3 and Figure 4).

### 3.6. Cox Proportional Hazards Regression Analysis

To address the potential temporal bias, we utilized a time-dependent Cox proportional hazards model. This approach accounts for the interval between admission and the first ketone measurement, ensuring that only patients at risk at the time of measurement were included. In fully adjusted models, urine ketone positivity in the ICU cohort remained independently associated with a reduced hazard of new-onset AF (HR 0.80, 95% CI 0.66–0.97, *p* = 0.023). Elevated serum β-hydroxybutyrate was likewise associated with a lower hazard of AF, although this association did not reach statistical significance (HR 0.39, 95% CI 0.10–1.49, *p* = 0.168), likely reflecting the limited sample size of the serum β-hydroxybutyrate cohort. In contrast, urine ketone positivity in the general ward cohort remained associated with an increased hazard of AF (HR 2.63, 95% CI 1.08–6.37, *p* = 0.032) (Table 10).

## 4. Discussion

Despite growing interest in ketone metabolism, the transition from a maladaptive metabolic byproduct to a cardioprotective substrate remains poorly understood. This study identified endogenous ketosis functions as a protective adaptive mechanism during acute illness while being associated with increased AF risk in lower-acuity general ward populations. In the ICU, ketone positivity was associated with a significant reduction in AF. In the serum β-hydroxybutyrate cohort, the multivariable model showed a trend toward a protective association (*p* = 0.053). After propensity score matching, elevated serum β-hydroxybutyrate concentrations were associated with approximately a 76% reduction in the odds of AF. Conversely, multivariable regression analyses demonstrated that ketone positivity was associated with a two-fold increase in AF risk in the general hospital ward. Subgroup analyses verified consistent directionality. Furthermore, urine ketone positivity was associated with lower mortality in both ICU and general ward cohorts, supporting the interpretation that endogenous ketosis reflects a metabolically adaptive phenotype rather than a marker of physiological deterioration. In contrast to AF outcomes, the association between serum β-OHB and mortality was attenuated after multivariable adjustment, suggesting that the mortality relationship may be more susceptible to confounding by illness severity and comorbidity burden. This divergence may also indicate a more direct mechanistic link between ketone metabolism and reduced arrhythmic susceptibility.

The observation of a protective association, despite a higher baseline comorbidity burden in ketone-positive patients, suggests that ketosis functions as an adaptive metabolic response to acute stress. Across both ICU and general ward cohorts, ketone positivity was associated with lower lactate concentrations, suggesting more efficient oxidative substrate utilization during acute illness. This metabolic profile was accompanied by reductions in systemic inflammatory indices, including white blood cell count, neutrophil-to-lymphocyte ratio, and red cell distribution width. Notably, insulin use was considerably more common than documented diabetes mellitus across both cohorts, suggestive of stress-induced hyperglycemia during acute illness. This physiological stress response promotes hepatic glucose production and peripheral insulin resistance, often necessitating exogenous insulin despite adequate endogenous insulin production. These findings suggest endogenous ketosis may reflect an adaptive metabolic response to critical illness, rather than solely diabetes-related dysfunctions. The observed association between ketosis and lower sepsis prevalence was consistent with a systemic protective metabolic profile. Stubbs et al. characterized β-hydroxybutyrate as an immunometabolic countermeasure that prevents maladaptive inflammatory responses without compromising immune integrity [30]. Given the central role of inflammation in atrial structural remodeling and electrophysiological instability during critical illness [31], attenuation of inflammatory signaling may represent an important link between endogenous ketosis and reduced atrial arrhythmogenic susceptibility during critical illness. This effect may be driven by β-hydroxybutyrylation of key signaling proteins, such as STAT1, which inhibits pro-inflammatory macrophage polarization [32].

Several biological factors may explain the observed association between ketone status and AF. The adult myocardium is characterized by high metabolic flexibility, primarily relying on fatty acid oxidation for ATP production [33]. However, in states of acute hemodynamic stress or critical illness, the failing heart undergoes a metabolic fuel shift [24]. Ketone bodies, specifically β-hydroxybutyrate, require less oxygen per mole of ATP produced compared to fatty acids [34]. By bypassing the complex β-oxidation pathway and entering the tricarboxylic acid (TCA) cycle directly via succinyl-CoA:3-oxoacid CoA-transferase (SCOT), ketones provide a more efficient energy source that may preserve myocardial electrophysiological stability during states of energy starvation and hypoxia.

**Figure 5:**
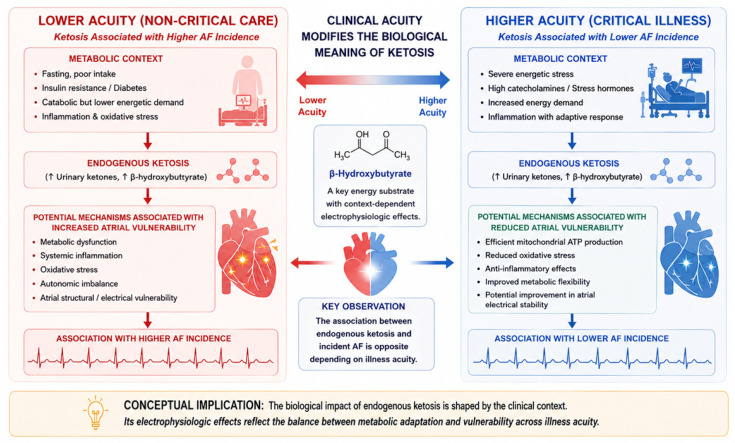
Proposed conceptual model of illness acuity-dependent associations between endogenous ketosis and atrial fibrillation. Red indicates a lower acuity (non-critical) clinical setting. Blue indicates a higher acuity (critical) clinical setting. Arrows illustrate the relationship between metabolic context, endogenous ketosis, and AF risk across different levels of illness acuity. Anatomical icons and ECG traces represent electric instability and AF incidence.

β-hydroxybutyrate has been shown to influence class I histone deacetylase activity and intracellular redox balance [35], suggesting that circulating ketone concentrations may directly influence atrial substrate vulnerability during acute illness. Furthermore, ketone metabolism actively enhances myocardial resilience through a process of mitohormesis [36], wherein nutritional ketosis has been reported to trigger adaptive mitochondrial signaling that bolsters antioxidant defenses and metabolic efficiency. Such enhancements are critical in the context of the stressed heart, where mitochondrial failure leads to the electrical instability and ion channel remodeling that underpin atrial arrhythmias [37]. Beyond energetic efficiency, ketones may directly modulate atrial electrophysiology. In experimental models of cardiac preservation, Seefeldt et al. suggest that β-hydroxybutyrate may stabilize the sarcolemma membrane by modulating ATP-sensitive potassium channels (K_ATP_), reducing oxidative stress-induced mitochondrial permeability transition pore opening [38]. By preserving mitochondrial integrity and reducing the production of reactive oxygen species, endogenous ketosis may reduce oxidative triggering of atrial premature beats that initiate AF (Figure 5).

The findings of the present study should be interpreted in light of several limitations. First, while the use of MIMIC-IV v3.1 provides a high-resolution clinical dataset, the retrospective observational design precludes the establishment of definitive causal inferences. Second, urinary measurements are semi-quantitative, and may not correspond with the total bioavailable ketone pool. Third, serum β-hydroxybutyrate measurements were available only in a subset of patients, which reduced statistical power and limited evaluation of potential dose–response relationships. Similarly, in both the multivariable model and the time-dependent Cox proportional hazards model, elevated serum β-hydroxybutyrate was associated with a low risk of AF; however, these associations did not reach statistical significance (Cox model: *p* = 0.168). Accordingly, the findings of this limited size cohort should be interpreted as an associative trend that require validation in future prospective studies. Fourth, differences between ICU and general ward populations may reflect underlying heterogeneity in illness acuity and metabolic state that cannot be fully accounted for in retrospective analyses. Fifth, while we utilized incident AF as a primary endpoint, the intermittent nature of paroxysmal AF in a hospital setting may lead to an underestimation of the true arrhythmic burden if episodes occurred between scheduled ECG monitoring or nursing assessments. Sixth, the time-dependent Cox proportional hazard model was utilized to mitigate temporal bias; however, residual confounding and unmeasured factors inherent to the retrospective study type may persist. Lastly, ketone measurements were not obtained routinely and were performed at the discretion of treating clinicians. Consequently, the study population was limited to patients who underwent urinary ketone or serum β-hydroxybutyrate assessment, which may limit the generalizability of the findings. Despite these limitations, this study represents one of the first large-scale analyses to compare urinary ketone status and circulating β-hydroxybutyrate concentrations across ICU and non-ICU cohorts.

Endogenous ketosis represents a marker of metabolic resilience in critically ill patients and a potential target for arrhythmia prevention. Since ketone metabolism can be modulated through dietary intervention, pharmacologic therapy, or exogenous ketone supplementation, the observed associations raise the possibility of a novel strategy for reducing AF in high-risk populations. The transition from observational association to therapeutic application is already underway. Ongoing trials, such as the KETO-AHF (NCT06653725) [39], are currently investigating the use of exogenous ketone esters and 1,3-butanediol to improve hemodynamic stability and reduce natriuretic peptides in acute heart failure patients. Ultimately, metabolic modulation via SGLT2 inhibitors or ketone supplementation may provide a ‘non-ion-channel’ dependent approach to rhythm management in the ICU.

Our findings align with a shifting paradigm in critical care nutrition and metabolic support. Current 2025 ELSO and ESICM guidelines emphasize metabolic flexibility and phase-specific nutritional strategies that avoid early overfeeding, which can suppress endogenous ketogenesis [40,41]. However, ketosis is still viewed through the lens of pathology (e.g., DKA), missing its potential role as an adaptive resilience marker in non-diabetic critical illness. Whereas diabetic ketoacidosis is characterized by uncontrolled hyperglycaemia, severe metabolic acidosis, and insulin deficiency; moderate elevations in circulating ketone bodies during critical illness often may reflect a compensatory shift toward more oxygen-efficient substrate utilization. Importantly, careful patient selection remains essential, particularly among individuals with diabetes mellitus or impaired insulin reserve, in whom the boundary between adaptive ketosis and ketoacidosis may be narrower.

## 5. Conclusions

This study shows that the association between endogenous ketosis and AF is modified by clinical acuity. In the ICU, elevated serum β-hydroxybutyrate demonstrated a trend toward reduced AF risk, while urine ketone positivity remained independently associated with a lower incidence of AF and lower mortality. These findings highlight ketone metabolism as a candidate mechanistic pathway in arrhythmia modulation and support the need for prospective studies to evaluate targeted metabolic interventions in high-acuity settings.

## Figures and Tables

**Table 1:** Definition of Ketosis Categories Based on Urine Ketone and Serum β-Hydroxybutyrate Measurements.

Metabolic State	Urine Ketones	Serum β-OHB (mmol/L)	Clinical Interpretation
No ketosis	Negative (<5 mg/dL)	<0.5	Predominant glucose utilization
Mild ketosis	Trace (5–20 mg/dL)	0.5–1.0	Early physiological ketosis (fasting or stress response)
Moderate ketosis	Small–moderate (20–40 mg/dL)	1.0–3.0	Nutritional or adaptive metabolic ketosis
Marked ketosis	Moderate–large (40–80 mg/dL)	3.0–5.0	Sustained ketone utilization during prolonged fasting or illness
Hyperketonemia	Large (>80 mg/dL)	>5.0	May indicate metabolic decompensation depending on glucose and acid–base status

**Table 2:** Patient Demographics and Baseline Characteristics for the ICU Cohort.

Category	Variable	Urine Ketones [−] [*n* = 2893]	Urine Ketones [+] [*n* = 7070]	*p*-Value	Serum β-OHB < 1.0 [*n* = 159]	Serum β-OHB ≥ 1.0 [*n* = 129]	*p*-Value
I. Patient Demographics	Age, years	62 [52–73]	63 [52–75]	0.014	59 [50–68]	60 [47–69]	0.747
	Body Mass Index	28.72 [24.57–34.16]	27.38 [23.47–32.26]	0.278	27.82 [23.23–36.04]	29.30 [24.11–35.57]	0.998
	Biological sex			0.231			0.469
	Female	1334 (46.1%)	3167 (44.8%)		77 (48.4%)	68 (52.7%)	
	Male	1559 (53.9%)	3903 (55.2%)		82 (51.6%)	61 (47.3%)	
II. Comorbidities, *n* (%)	Hypertension	516 (17.8%)	1307 (18.5%)	0.458	50 (31.4%)	47 (36.4%)	0.383
	Diabetes Mellitus	413 (14.3%)	1019 (14.4%)	0.875	96 (60.4%)	103 (79.8%)	<0.001
	Heart Failure	292 (10.1%)	629 (8.9%)	0.060	34 (21.4%)	20 (15.5%)	0.204
	Prior MI	182 (6.3%)	480 (6.8%)	0.376	32 (20.1%)	23 (17.8%)	0.622
	COPD	164 (5.7%)	377 (5.3%)	0.498	16 (10.1%)	10 (7.8%)	0.541
	Stroke	72 (2.5%)	229 (3.2%)	0.053	8 (5.0%)	7(5.4%)	>0.999
	OSAS	135 (4.7%)	323 (4.6%)	0.829	16 (10.1%)	14 (10.8%)	0.848
	Renal Insufficiency	275 (9.5%)	741 (10.5%)	0.146	50 (31.4%)	31 (24.0%)	0.164
	Oncology	380 (13.1%)	758 (10.7%)	<0.001	21 (13.2%)	19 (14.7%)	0.734
	Sepsis	983 (34.0%)	1729 (24.5%)	<0.001	57 (35.8%)	30 (23.3%)	0.021
	DKA	37 (1.3%)	155 (2.2%)	0.003	27 (17.0%)	71 (55.0%)	<0.001
III. Clinical Indices	Charlson Comorbidity Index *	1.84 ± 1.42	1.78 ± 1.54	0.086	2.23 ± 1.41	2.09 ± 1.43	0.407
	CHA_2_DS_2_–VASc *	2.22 ± 1.57	2.35 ± 1.58	<0.001	2.33 ± 1.39	2.60 ± 1.33	0.102
IV. Medications	Insulin Use	1860 (64.3%)	4566 (64.6%)	0.784	137 (86.2%)	118 (91.5%)	0.160
	Beta–Blocker Use	1046 (36.2%)	2762 (39.1%)	0.007	38 (23.9%)	32 (24.8%)	0.858
	Anti–arrhythmic use	142 (4.9%)	267 (3.8%)	0.010	8 (5.0%)	1 (0.8%)	0.039
V. Vitals	Mean HR, bpm	87.07 [77.19–97.71]	84.99 [75.23–94.92]	<0.001	86.12 [78.14–98.36]	89.40 [77.47–96.89]	0.545
	Mean BP, mmHg	77.73 [71.12–85.50]	81.87 [74.94–90.65]	<0.001	77.16 [71.45–86.18]	80.20 [74.54–87.48]	0.257
	SpO_2_, %	96.52 [95.24–97.65]	96.78 [95.54–97.90]	<0.001	97.02 [95.67–97.98]	96.80 [95.86–98.03]	0.612
	RR Variability	9.66 [6.80–12.95]	9.53 [7.01–12.77]	0.939	9.73 [7.03–13.29]	8.95 [6.61–13.34]	0.392
VI. Metabolic	Glucose, mmol/L	10.30 [8.90–11.80]	10.70 [9.20–12.30]	0.041	10.05 [8.60–11.30]	11.40 [8.30–13.40]	0.180
	Lactate, mmol/L	2.90 [1.80–4.90]	2.20 [1.50–3.80]	<0.001	3.65 [2.00–7.30]	2.20 [1.40–4.20]	<0.001
	Uric Acid, mg/dL	7.50 [4.80–10.00]	5.90 [3.80–8.70]	<0.001	7.05 [5.45–9.65]	9.50 [5.30–13.50]	0.355
	Phosphate, mg/dL	5.10 [4.20–6.60]	4.70 [4.00–5.80]	<0.001	5.60 [4.50–7.70]	4.55 [3.80–5.80]	<0.001
VII. Inflammatory	WBC, ×10^9^/L	17.50 [12.50–24.20]	15.10 [10.90–21.00]	<0.001	17.60 [12.20–27.30]	15.75 [11.15–21.85]	0.026
	NLR	6.73 [4.14–11.00]	5.52 [3.45–9.23]	<0.001	5.52 [3.12–9.37]	5.37 [3.29–10.05]	0.888
	RDW, %	17.10 [15.00–20.20]	15.80 [14.30–18.00]	<0.001	17.20 [14.60–21.40]	14.60 [13.40–16.25]	<0.001
	CRP, mg/L	90.10 [46.40–184.60]	95.15 [32.70–174.40]	0.187	114.55 [47.15–174.90]	94.10 [15.10–142.30]	0.379
VIII. Organ Function	Creatinine, mg/dL	1.90 [1.20–3.20]	1.60 [1.00–2.80]	<0.001	2.30 [1.30–4.40]	1.40 [1.00–2.50]	<0.001
	BUN, mg/dL	47.00 [27.00–76.00]	34.00 [21.00–60.00]	<0.001	48.00 [26.00–94.00]	30.00 [17.00–64.00]	<0.001
	BUN/Cr Ratio	22.86 [16.67–30.83]	20.63 [15.45–27.78]	<0.001	19.14 [13.70–26.74]	18.57 [13.54–24.17]	0.298
	LDH, U/L	365.00 [259.00–619.00]	333.00 [233.00–549.00]	<0.001	424.00 [263.50–739.50]	330.00 [230.00–601.00]	0.065
	Albumin, g/dL	3.30 [2.80–3.80]	3.30 [2.90–3.70]	0.877	3.40 [2.90–4.00]	3.50 [3.00–3.90]	0.636
	BNP, pg/mL	2740.00 [847.00–9351.00]	2249.50 [646.00–8023.00]	0.003	2184.00 [703.00–4031.00]	3030.00 [885.00–8696.00]	0.257
	Troponin–T, ng/mL	0.09 [0.04–0.44]	0.10 [0.04–0.40]	0.035	0.11 [0.04–0.44]	0.16 [0.04–0.64]	0.372
IX. Electrolytes	HCO_3_^−^, mEq/L	19.00 [16.00–22.00]	18.00 [16.00–21.00]	<0.001	21.00 [17.00–26.00]	23.00 [20.00–27.00]	0.015
	Potassium, mEq/L	5.00 [4.60–5.70]	4.90 [4.50–5.50]	<0.001	5.20 [4.70–6.20]	5.10 [4.70–5.90]	0.500
	Calcium, mg/dL	9.10 [8.60–9.70]	9.20 [8.70–9.70]	0.515	9.50 [9.00–10.20]	9.20 [8.80–9.70]	0.001
X. Haematology	Platelets, ×10^3^/μL	270.00 [168.00–397.00]	302.00 [214.00–434.00]	<0.001	285.00 [204.00–412.00]	281.00 [224.50–412.00]	0.488
	Hemoglobin, g/dL	9.30 [8.40–10.60]	9.70 [8.60–11.10]	<0.001	9.10 [7.90–10.90]	10.70 [9.10–12.05]	<0.001
	Hematocrit, %	28.40 [25.70–32.30]	29.40 [26.50–33.70]	<0.001	28.50 [25.20–34.10]	32.95 [28.30–36.50]	<0.001
XI. Clinical Management & Interventions	Mechanical Ventilation	1265 (43.7%)	2797 (39.6%)	<0.001	28 (17.6%)	15 (11.6%)	0.157
	Vasopressor Support	1090 (37.7%)	2127 (30.1%)	<0.001	31 (19.5%)	11 (8.5%)	0.009
	Renal Replacement Therapy	272 (9.4%)	453 (6.4%)	<0.001	27 (17.0%)	7 (5.4%)	0.003
	Total Parenteral Nutrition	124 (4.3%)	226 (3.2%)	0.007	2 (1.3%)	0 (0.0%)	0.201
	Enteral Nutrition Status	45 (1.6%)	60 (0.9%)	0.002	0 (0.0%)	0 (0.0%)	N/A
XII. Clinical Service Category	Medical	1887 (73.6%)	3985 (67.2%)	<0.001	118 (85.5%)	95 (81.9%)	0.436
	Surgical	677 (26.4%)	1942 (32.8%)		20 (14.5%)	21 (18.1%)	

* Reported as Mean ± SD to illustrate directional trends in score distribution; all other continuous variables are reported as Median [IQR]. Continuous variables were compared using independent *t* tests or Mann–Whitney U tests as appropriate, and categorical variables using χ^2^ or Fisher’s exact tests. N/A, not applicable; due to insufficient data for statistical comparison. Abbreviations: β-OHB, β-hydroxybutyrate; BNP, B type natriuretic peptide; BP, blood pressure; bpm, beats per minute; BUN, blood urea nitrogen; CHA_2_DS_2_–VASc, congestive heart failure, hypertension, age ≥75 years, diabetes mellitus, stroke, vascular disease, age 65–74 years, sex category; COPD, chronic obstructive pulmonary disease; CRP, C reactive protein; Cr, creatinine; DKA, diabetic ketoacidosis; HR, heart rate; LDH, lactate dehydrogenase; MI, myocardial infarction; NLR, neutrophil to lymphocyte ratio; OSAS, obstructive sleep apnea syndrome; RDW, red cell distribution width; RR variability, respiratory rate variability; SpO_2_, peripheral oxygen saturation; WBC, white blood cell count.

**Table 3:** Patient Demographics and Baseline Characteristics for the General Ward Cohort.

Category	Variable	Urine Ketones [−] [*n* = 3159]	Urine Ketones [+] [*n* = 10,191]	*p*-Value	Serum β-OHB < 1.0 [*n* = 171]	Serum β-OHB ≥ 1.0 [*n* = 120]	*p*-Value
I. Patient Demographics	Age, years	62 [51–73]	64 [52–76]	<0.001	60 [44–70]	56 [36–65]	0.027
	Body Mass Index	29.03 [24.85–34.49]	27.40 [23.55–32.27]	0.306	27.45 [22.01–35.81]	28.62 [22.17–32.90]	0.483
	Biological sex			<0.001			0.436
	Female	1550 (49.1%)	4594 (45.1%)		79 (46.2%)	61 (50.8%)	
	Male	1609 (50.9%)	5597 (54.9%)		92 (53.8%)	59 (49.2%)	
II. Comorbidities, *n* (%)	Hypertension	1251 (39.6%)	4129 (40.5%)	0.365	53 (31.0%)	42 (35.0%)	0.473
	Diabetes Mellitus	766 (24.3%)	2937 (28.8%)	<0.001	82 (48.0%)	58 (48.3%)	0.949
	Heart Failure	558 (17.7%)	1527 (15.0%)	<0.001	29 (17.0%)	14 (11.7%)	0.210
	Prior MI	312 (9.9%)	987 (9.7%)	0.748	24 (14.0%)	14 (11.7%)	0.555
	COPD	297 (9.4%)	903 (8.9%)	0.351	13 (7.6%)	8 (6.7%)	0.761
	Stroke	90 (2.9%)	391 (3.8%)	0.027	9 (5.3%)	6 (5.0%)	>0.999
	OSAS	201 (6.4%)	621 (6.1%)	0.580	13 (7.6%)	8 (6.7%)	0.761
	Renal Insufficiency	524 (16.6%)	2546 (25.0%)	<0.001	47 (27.5%)	23 (19.2%)	0.102
	Oncology	725 (22.9%)	2286 (22.4%)	0.538	26 (15.2%)	15 (12.5%)	0.514
	Sepsis	345 (10.9%)	700 (6.9%)	<0.001	46 (26.9%)	16 (13.3%)	0.005
	DKA	24 (0.8%)	120 (1.2%)	0.047	19 (11.1%)	59 (49.2%)	<0.001
III. Clinical Indices	Charlson Comorbidity Index *	2.72 ± 2.12	3.03 ± 2.22	<0.001	2.95 ± 2.16	2.37 ± 2.11	0.021
	CHA_2_DS_2_–VASc *	2.07 ± 1.48	2.18 ± 1.47	<0.001	2.09 ± 1.46	1.94 ± 1.37	0.366
IV. Medications	Insulin Use	1557 (49.3%)	5097 (50.0%)	0.475	124 (72.5%)	98 (81.7%)	0.071
	Beta–Blocker Use	1005 (31.8%)	3633 (35.6%)	<0.001	44 (25.7%)	28 (23.3%)	0.641
	Anti–arrhythmic use	92 (2.9%)	186 (1.8%)	<0.001	6 (3.5%)	1 (0.8%)	0.143
V. Vitals	Mean HR, bpm	86.96 [77.60–96.65]	85.19 [75.64–94.70]	<0.001	85.66 [79.09–97.02]	88.80 [79.65–96.85]	0.433
	Mean BP, mmHg	76.90 [70.69–84.35]	81.73 [74.84–89.90]	<0.001	82.11 [74.32–89.43]	85.04 [80.72–91.73]	0.014
	SpO_2_, %	96.51 [95.26–97.58]	96.84 [95.66–97.92]	<0.001	96.98 [95.84–97.94]	96.85 [95.93–97.95]	0.776
	RR Variability	10.31 [7.42–13.40]	10.04 [7.50–13.21]	0.453	12.07 [8.32–14.09]	10.25 [6.65–13.24]	0.054
VI. Metabolic	Glucose, mmol/L	9.25 [8.30–10.99]	9.91 [8.67–11.50]	<0.001	10.63 [8.25– 11.95]	12.00 [10.58– 12.10]	0.151
	Lactate, mmol/L	1.86 [1.35–2.58]	1.55 [1.15–2.15]	<0.001	1.90 [1.47–2.90]	1.60 [1.10–2.05]	<0.001
	Uric Acid, mg/dL	5.74 [4.00–8.40]	4.90 [3.37–7.20]	<0.001	5.17 [4.35–7.03]	6.72 [5.30–11.60]	0.154
	Phosphate, mg/dL	3.38 [2.94–3.95]	3.41 [2.98–3.93]	0.234	3.59 [3.02–4.31]	3.08 [2.69–3.59]	<0.001
VII. Inflammatory	WBC, ×10^9^/L	10.54 [7.65–13.78]	9.02 [6.72–11.97]	<0.001	9.21 [7.10–12.70]	9.20 [7.10–12.42]	0.882
	NLR	7.59 [4.49–12.32]	5.73 [3.29–9.78]	<0.001	5.84 [3.44–11.32]	5.76 [3.74–10.51]	0.670
	RDW, %	15.07 [13.77–17.18]	14.35 [13.37–15.76]	<0.001	14.79 [13.20–17.67]	13.82 [12.68–14.74]	<0.001
	CRP, mg/L	80.00 [33.80–141.30]	62.13 [18.80–123.10]	0.005	68.50 [24.66–160.00]	62.05 [21.70–115.27]	0.595
VIII. Organ Function	Creatinine, mg/dL	1.13 [0.77–1.72]	1.14 [0.76–1.71]	<0.001	1.17 [0.75–2.23]	0.88 [0.66–1.43]	<0.001
	BUN, mg/dL	23.50 [14.57–40.29]	20.50 [13.20–34.25]	<0.001	22.47 [14.27–44.24]	16.81 [10.40–30.34]	<0.001
	BUN/Cr Ratio	20.12 [15.17–26.97]	17.92 [13.60–23.33]	<0.001	18.18 [13.17–25.35]	15.81 [12.66–21.13]	0.027
	LDH, U/L	283.00 [209.00–417.00]	258.05 [197.00–374.50]	<0.001	337.00 [214.00–526.68]	320.00 [223.50–430.50]	0.336
	Albumin, g/dL	2.98 [2.57–3.40]	3.17 [2.73–3.60]	<0.001	3.15 [2.64–3.70]	3.40 [2.85–3.80]	0.026
	BNP, pg/mL	2062 [600–6558]	1782 [494.5–5471.5]	0.214	2622 [703–7900]	2236 [602–11,332]	0.775
	Troponin–T, ng/mL	0.09 [0.03–0.42]	0.09 [0.03–0.35]	0.016	0.12 [0.04–0.61]	0.16 [0.04–0.64]	0.758
IX. Electrolytes	HCO_3_^−^, mEq/L	24 [21.65–26.24]	24.25 [22.06–26.40]	<0.001	21.49 [19.13–24.27]	20.56 [18.29–23.00]	0.007
	Potassium, mEq/L	4.07 [3.82– 4.35]	4.09 [3.83–4.39]	0.012	4.11 [3.90–4.45]	4.05 [3.78–4.32]	0.051
	Calcium, mg/dL	8.45 [8.06–8.88]	8.60 [8.20–8.99]	<0.001	8.73 [8.18–9.12]	8.70 [8.32–8.99]	0.644
X. Haematology	Platelets, ×10^3^/μL	206 [125.13–290.55]	228 [166.75–302.33]	<0.001	221.75 [157.64–279.12]	227.00 [178.00–291.00]	0.207
	Hemoglobin, g/dL	9.80 [8.60–11.30]	10.40 [9.00–11.80]	<0.001	10.60 [8.90–12.45]	11.15 [10.00–12.30]	0.070
	Hematocrit, %	29.60 [26.30–33.90]	31.20 [27.50–35.40]	<0.001	32.95 [27.45–37.75]	33.50 [30.90–37.35]	0.127
XI. Clinical Management & Interventions	Renal Replacement Therapy	14 (0.4%)	72 (0.7%)	0.107	6 (4.8%)	3 (2.7%)	0.409
	Total Parenteral Nutrition	81 (2.6%)	175 (1.7%)	0.002	1 (0.8%)	0 (0.0%)	0.347
	Enteral Nutrition Status	36 (1.1%)	44 (0.4%)	<0.001	0 (0.0%)	0 (0.0%)	N/A
XII. Clinical Service Category	Medical	2276 (72.0%)	8050 (79.0%)	<0.001	137 (80.1%)	93 (77.5%)	0.589
	Surgical	883 (28.0%)	2141 (21.0%)		34 (19.9%)	27 (22.5%)	

* Reported as Mean ± SD to illustrate directional trends in score distribution; all other continuous variables are reported as Median [IQR]. Continuous variables were compared using independent *t* tests or Mann–Whitney U tests as appropriate, and categorical variables using χ^2^ or Fisher’s exact tests. N/A, not applicable; due to insufficient data for statistical comparison. Abbreviations: β-OHB, β-hydroxybutyrate; BNP, B type natriuretic peptide; BP, blood pressure; bpm, beats per minute; BUN, blood urea nitrogen; CHA_2_DS_2_–VASc, congestive heart failure, hypertension, age ≥75 years, diabetes mellitus, stroke, vascular disease, age 65–74 years, sex category; COPD, chronic obstructive pulmonary disease; CRP, C reactive protein; Cr, creatinine; DKA, diabetic ketoacidosis; HR, heart rate; LDH, lactate dehydrogenase; MI, myocardial infarction; NLR, neutrophil to lymphocyte ratio; OSAS, obstructive sleep apnea syndrome; RDW, red cell distribution width; RR variability, respiratory rate variability; SpO_2_, peripheral oxygen saturation; WBC, white blood cell count.

**Table 4:** Overall Incidence of AF and All-Cause Mortality.

Cohort	*n*	AF *n* [%]	All-Cause Mortality *n* [%]
ICU	10,251	582 (5.7%)	1814 (17.7%)
General ward	13,641	113 (0.8%)	1243 (9.1%)

Data are presented as *n* (%). Abbreviations: AF, Atrial Fibrillation; ICU, intensive care unit.

**Table 5:** Incidence of AF and Mortality by Ketone Status.

Cohort	Ketone Marker	Group	*n*	AF *n* [%]	*p*-Value	All-Cause Mortality *n* [%]	*p*-Value
ICU	Urine ketones	Negative	2893	198 [6.8%]		790 [27.3%]	
		Positive	7070	365 [5.2%]	0.001	981 [13.9%]	<0.001
	Serum β-OHB	Low [<1.0 mmol/L]	159	15 [9.4%]		30 [18.9%]	
		High [≥1.0 mmol/L]	129	4 [3.1%]	0.034	13 [10.1%]	0.037
General ward	Urine ketones	Negative	3159	16 [0.5%]		530 [16.8%]	
		Positive	10,191	96 [0.9%]	0.019	684 [6.7%]	<0.001
	Serum β-OHB	Low [<1.0 mmol/L]	171	1 [0.6%]		23 [13.5%]	
		High [≥1.0 mmol/L]	120	0 [0.0%]	0.401	6 [5.0%]	0.018

Data are presented as *n* (%). Abbreviations: AF, Atrial Fibrillation; β-OHB, β-hydroxybutyrate; ICU, intensive care unit.

**Table 6:** Multivariable Logistic Regression Models Evaluating the Association Between Ketone Status and AF in ICU and General ward Cohorts.

Cohort	Ketone Marker	Adjustment Model	Variables Included	Odds Ratio [95% CI]	*p*-Value
ICU	Urine Ketones	Model 1	Unadjusted	0.74 [0.62–0.89]	0.001
		Model 2	Age, sex	0.70 [0.59–0.84]	<0.001
		Model 3	Model 2 + comorbidities and medications	0.71 [0.59–0.85]	<0.001
		Model 4	Model 3 + inflammatory status	0.79 [0.64–0.98]	0.032
ICU	Serum Β-OHB	Model 1	Unadjusted	0.31 [0.10–0.95]	0.040
		Model 2	Age, sex	0.31 [0.10–0.98]	0.045
		Model 3	Model 2 + comorbidities and medications	0.35 [0.11–1.13]	0.079
		Model 4	Model 3 + inflammatory status	0.25 [0.06–1.02]	0.053
General ward	Urine Ketones	Model 1	Unadjusted	1.87 [1.10–3.18]	0.021
		Model 2	Age, sex	1.70 [1.00–2.90]	0.051
		Model 3	Model 2 + comorbidities	2.55 [1.41–4.61]	0.002
		Model 4	Model 3 + inflammatory status	2.62 [1.03–6.66]	0.044

Model 1: Unadjusted. Model 2: Adjusted for age and biological sex. Model 3: Adjusted for age and biological sex, hypertension, diabetes mellitus, heart failure, prior MI, stroke, COPD, renal insufficiency, oncology, exogenous insulin, beta-blocker use and anti-arrhythmic use. Model 4: Adjusted for age and biological sex, hypertension, diabetes mellitus, heart failure, prior MI, stroke, COPD, renal insufficiency, oncology, exogenous insulin, beta-blocker use, anti-arrhythmic use and NLR.

**Table 7:** Multivariable Logistic Regression Models Evaluating the Association Between Ketone Status and All-Cause Mortality in ICU and General ward Cohorts.

Cohort	Ketone Marker	Adjustment Model	Variables Included	Odds Ratio [95% CI]	*p*-Value
ICU	Urine Ketones	Model 1	Unadjusted	0.43 [0.39–0.48]	<0.001
		Model 2	Age, sex	0.42 [0.38–0.47]	<0.001
		Model 3	Model 2 + comorbidities and medications	0.43 [0.39–0.48]	<0.001
		Model 4	Model 3 + inflammatory status	0.44 [0.39–0.50]	<0.001
ICU	Serum β-OHB	Model 1	Unadjusted	0.48 [0.24–0.97]	0.040
		Model 2	Age, sex	0.48 [0.24–0.98]	0.043
		Model 3	Model 2 + comorbidities and medications	0.54 [0.24–1.20]	0.130
		Model 4	Model 3 + inflammatory status	0.62 [0.25–1.54]	0.306
General ward	Urine Ketones	Model 1	Unadjusted	0.36 [0.32–0.40]	<0.001
		Model 2	Age, sex	0.35 [0.31–0.40]	<0.001
		Model 3	Model 2 + comorbidities	0.36 [0.32–0.41]	<0.001
		Model 4	Model 3 + inflammatory status	0.41 [0.35–0.47]	<0.001
General ward	Serum β-OHB	Model 1	Unadjusted	0.34 [0.13–0.86]	0.023
		Model 2	Age, sex	0.39 [0.15–1.00]	0.049
		Model 3	Model 2 + comorbidities	0.44 [0.16–1.19]	0.105
		Model 4	Model 3 + inflammatory status	0.47 [0.14–1.65]	0.242

Model 1: Unadjusted. Model 2: Adjusted for age and biological sex. Model 3: Adjusted for age and biological sex, hypertension, diabetes mellitus, heart failure, prior MI, stroke, COPD, renal insufficiency, oncology, exogenous insulin, beta-blocker use and anti-arrhythmic use. Model 4: Adjusted for age and biological sex, hypertension, diabetes mellitus, heart failure, prior MI, stroke, COPD, renal insufficiency, oncology, exogenous insulin, beta-blocker use, anti-arrhythmic use and NLR.

**Table 8:** Propensity Score–Matched Estimates of the Association Between Ketone Status and AF in ICU and General ward Cohorts.

Cohort	Ketone	Odds Ratio [95% CI]	*p*-Value
ICU	Urine	0.68 [0.52–0.88]	0.004
ICU	Serum β-OHB *	0.24 [0.08–0.70]	0.003
General ward	Urine	2.88 [0.89–9.29]	0.077

Propensity scores were estimated using a logistic regression model including age, sex, comorbidities, medications, and NLR. * Anti-arrhythmic medication use was not included in serum β-hydroxybutyrate models due to limited event counts.

**Table 9:** Propensity Score–Matched Estimates of the Association Between Ketone Status and All-Cause Mortality in ICU and General ward Cohorts.

Cohort	Ketone	Odds Ratio [95% CI]	*p*-Value
ICU	Urine	0.53 [0.42–0.65]	<0.001
	Serum β-OHB *	0.79 [0.26–2.44]	0.687
General ward	Urine	0.38 [0.34–0.42]	<0.001
	Serum β-OHB *	0.64 [0.13–3.24]	0.590

Propensity scores were estimated using a logistic regression model including age, sex, comorbidities, medications, and NLR. * Anti-arrhythmic medication use was not included in serum β-hydroxybutyrate models due to limited event counts.

**Table 10:** Time-Dependent Cox Proportional Hazards Analysis.

Cohort	Ketone	Adjusted HR [95% CI]	*p*-Value
ICU	Urine	0.80 [0.66–0.97]	0.023
	Serum β-OHB *	0.39 [0.10–1.49]	0.168
General ward	Urine	2.63 [1.08–6.37]	0.032

Adjusted for age, sex, hypertension, diabetes mellitus, heart failure, prior myocardial infarction, stroke, COPD, renal insufficiency, oncology, insulin use, beta-blocker use, anti-arrhythmic use, and NLR. * Anti-arrhythmic medication use was not included in serum β-hydroxybutyrate models due to limited event counts.

## Data Availability

All data analyzed in this study are available through the MIMIC-IV database.

## Abbreviations

The following abbreviations are used in this manuscript:

AF	Atrial fibrillation
ATET	Average treatment effect on the treated
ATP	Adenosine triphosphate
β-OHB	β-Hydroxybutyrate
BNP	B-type natriuretic peptide
BUN	Blood urea nitrogen
CCI	Charlson Comorbidity Index
CHA_2_DS_2_-VASc	Congestive heart failure, Hypertension, Age ≥75 years, Diabetes mellitus, Stroke/TIA/thromboembolism, Vascular disease, Age 65–74 years, Sex category
CI	Confidence interval
CITI	Collaborative Institutional Training Initiative
COPD	Chronic obstructive pulmonary disease
CRP	C-reactive protein
DKA	Diabetic ketoacidosis
ECG	Electrocardiogram
ELSO	Extracorporeal Life Support Organization
ESICM	European Society of Intensive Care Medicine
HDAC	Histone deacetylase
HIPAA	Health Insurance Portability and Accountability Act
ICU	Intensive care unit
IQR	Interquartile range
LDH	Lactate dehydrogenase
MAP	Mean arterial pressure
MIMIC-IV	Medical Information Mart for Intensive Care IV
mPTP	Mitochondrial permeability transition pore
NLR	Neutrophil-to-lymphocyte ratio
OR	Odds ratio
RDW	Red cell distribution width
ROS	Reactive oxygen species
SCOT	Succinyl-CoA:3-oxoacid CoA-transferase
SpO_2_	Peripheral oxygen saturation
SQL	Structured query language
STAT1	Signal transducer and activator of transcription 1
STROBE	Strengthening the Reporting of Observational Studies in Epidemiology
TCA	Tricarboxylic acid cycle

## References

[1] Bosch N.A., Cimini J., Walkey A.J. (2018). Atrial Fibrillation in the ICU. Chest.

[2] Corica B., Tartaglia F., Oliva A., Raparelli V., Cangemi R., Basili S., Lip G.Y.H., Proietti M., Romiti G.F. (2023). Prevalence of New-Onset Atrial Fibrillation in Hospitalized Patients with Community-Acquired Pneumonia: A Systematic Review and Meta-Analysis. Intern. Emerg. Med..

[3] Bedford J.P., Ferrando-Vivas P., Redfern O., Rajappan K., Harrison D.A., Watkinson P.J., Doidge J.C. (2022). New-Onset Atrial Fibrillation in Intensive Care: Epidemiology and Outcomes. Eur. Heart J. Acute Cardiovasc. Care.

[4] Wetterslev M., Haase N., Hassager C., Belley-Cote E.P., Mcintyre W.F., An Y., Shen J., Cavalcanti A.B., Zampieri F.G., Guimaraes H.P. (2019). New-Onset Atrial Fibrillation in Adult Critically Ill Patients: A Scoping Review. Intensive Care Med..

[5] Walkey A.J., Hammill B.G., Curtis L.H., Benjamin E.J. (2014). Long-Term Outcomes Following Development of New-Onset Atrial Fibrillation during Sepsis. Chest.

[6] Lopaschuk G.D., Karwi Q.G., Tian R., Wende A.R., Abel E.D. (2021). Cardiac Energy Metabolism in Heart Failure. Circ. Res..

[7] Wang L., Chen P., Xiao W. (2021). β-Hydroxybutyrate as an Anti-Aging Metabolite. Nutrients.

[8] Nielsen R., Møller N., Gormsen L.C., Tolbod L.P., Hansson N.H., Sorensen J., Harms H.J., Frøkiær J., Eiskjaer H., Jespersen N.R. (2019). Cardiovascular Effects of Treatment With the Ketone Body 3-Hydroxybutyrate in Chronic Heart Failure Patients. Circulation.

[9] Yurista S.R., Nguyen C.T., Rosenzweig A., de Boer R.A., Westenbrink B.D. (2021). Ketone Bodies for the Failing Heart: Fuels That Can Fix the Engine?. Trends Endocrinol. Metab..

[10] Umpierrez G., Korytkowski M. (2016). Diabetic Emergencies—Ketoacidosis, Hyperglycaemic Hyperosmolar State and Hypoglycaemia. Nat. Rev. Endocrinol..

[11] Preiser J.-C., Ichai C., Orban J.-C., Groeneveld A.B.J. (2014). Metabolic Response to the Stress of Critical Illness. Br. J. Anaesth..

[12] Singer P., Blaser A.R., Berger M.M., Calder P.C., Casaer M., Hiesmayr M., Mayer K., Montejo-Gonzalez J.C., Pichard C., Preiser J.-C. (2023). ESPEN Practical and Partially Revised Guideline: Clinical Nutrition in the Intensive Care Unit. Clin. Nutr..

[13] Glatz J.F.C., Nabben M., Young M.E., Schulze P.C., Taegtmeyer H., Luiken J.J.F.P. (2020). Re-Balancing Cellular Energy Substrate Metabolism to Mend the Failing Heart. Biochim. Biophys. Acta (BBA) Mol. Basis Dis..

[14] Grigoriou K., Karakasis P., Theofilis P., Vlachakis P.K., Milaras N., Patoulias D., Antoniadis A.P., Fragakis N. (2025). Metabolic Remodeling and Mitochondrial Stress in Atrial Fibrillation: Mechanisms and Translational Targets. Rev. Cardiovasc. Med..

[15] Bode D., Pronto J.R.D., Schiattarella G.G., Voigt N. (2024). Metabolic Remodelling in Atrial Fibrillation: Manifestations, Mechanisms and Clinical Implications. Nat. Rev. Cardiol..

[16] Balan A.I., Halațiu V.B., Scridon A. (2024). Oxidative Stress, Inflammation, and Mitochondrial Dysfunction: A Link between Obesity and Atrial Fibrillation. Antioxidants.

[17] Pfenniger A., Yoo S., Arora R. (2024). Oxidative Stress and Atrial Fibrillation. J. Mol. Cell. Cardiol..

[18] Attia A., Muthukumarasamy K.M., Al-U’Datt D.G.F., Hiram R. (2025). Relevance of Targeting Oxidative Stress, Inflammatory, and Pro-Resolution Mechanisms in the Prevention and Management of Postoperative Atrial Fibrillation. Antioxidants.

[19] Manolis A.S., Manolis T.A., Manolis A.A. (2023). Ketone Bodies and Cardiovascular Disease: An Alternate Fuel Source to the Rescue. Int. J. Mol. Sci..

[20] Chu Y., Zhang C., Xie M. (2021). Beta-Hydroxybutyrate, Friend or Foe for Stressed Hearts. Front. Aging.

[21] Tang M., Tu Y., Gong Y., Yang Q., Wang J., Zhang Z., Qin J., Niu S., Yi J., Shang Z. (2025). β-Hydroxybutyrate Facilitates Mitochondrial-Derived Vesicle Biogenesis and Improves Mitochondrial Functions. Mol. Cell.

[22] Lee T.-I., Trang N.N., Lee T.-W., Higa S., Kao Y.-H., Chen Y.-C., Chen Y.-J. (2023). Ketogenic Diet Regulates Cardiac Remodeling and Calcium Homeostasis in Diabetic Rat Cardiomyopathy. Int. J. Mol. Sci..

[23] Matsuura T.R., Puchalska P., Crawford P.A., Kelly D.P. (2023). Ketones and the Heart: Metabolic Principles and Therapeutic Implications. Circ. Res..

[24] Aubert G., Martin O.J., Horton J.L., Lai L., Vega R.B., Leone T.C., Koves T., Gardell S.J., Krüger M., Hoppel C.L. (2016). The Failing Heart Relies on Ketone Bodies as a Fuel. Circulation.

[25] St-Pierre V., Richard G., Croteau E., Fortier M., Vandenberghe C., Carpentier A.C., Cuenoud B., Cunnane S.C. (2024). Cardiorenal Ketone Metabolism in Healthy Humans Assessed by (11)C-Acetoacetate PET: Effect of D-β-Hydroxybutyrate, a Meal, and Age. Front. Physiol..

[26] Yoo B.M., Kim S.R., Lee B.-W. (2025). Ketone Body Induction: Insights into Metabolic Disease Management. Biomedicines.

[27] Wei S., Binbin L., Yuan W., Zhong Z., Donghai L., Caihua H. (2022). β-Hydroxybutyrate in Cardiovascular Diseases: A Minor Metabolite of Great Expectations. Front. Mol. Biosci..

[28] Chevli P.A., Mirzai S., Kazibwe R., Kingsley J., Wood A.C., Yeboah J., Slipczuk L., Mehta A., Bhatia H.S., Pandey A. (2026). Circulating Ketone Bodies and Incident Cardiovascular Outcomes and Mortality: Insights From the UK Biobank. J. Am. Heart Assoc..

[29] Johnson A.E.W., Bulgarelli L., Shen L., Gayles A., Shammout A., Horng S., Pollard T.J., Hao S., Moody B., Gow B. (2023). MIMIC-IV, a Freely Accessible Electronic Health Record Dataset. Sci. Data.

[30] Stubbs B.J., Koutnik A.P., Goldberg E.L., Upadhyay V., Turnbaugh P.J., Verdin E., Newman J.C. (2020). Investigating Ketone Bodies as Immunometabolic Countermeasures against Respiratory Viral Infections. Med.

[31] Scott L.J., Li N., Dobrev D. (2019). Role of Inflammatory Signaling in Atrial Fibrillation. Int. J. Cardiol..

[32] Bai Y.-P., Xing Y.-J., Ma T., Li K., Zhang T., Wang D.-G., Wan S.-J., Zhang C.-W., Sun Y., Wang M.-Y. (2024). β-Hydroxybutyrate Suppresses M1 Macrophage Polarization through β-Hydroxybutyrylation of the STAT1 Protein. Cell Death Dis..

[33] Actis Dato V., Lange S., Cho Y. (2024). Metabolic Flexibility of the Heart: The Role of Fatty Acid Metabolism in Health, Heart Failure, and Cardiometabolic Diseases. Int. J. Mol. Sci..

[34] Papazafiropoulou A.K., Georgopoulos M.M., Katsilambros N.L. (2021). Ketone Bodies and the Heart. Arch. Med. Sci. Atheroscler. Dis..

[35] Pali D.V., Kim S., Mantik K.E.K., Lee J.-B., So C.-Y., Moon S., Park D.-H., Kwak H.-B., Kang J.-H. (2025). Unraveling the Translational Relevance of β-Hydroxybutyrate as an Intermediate Metabolite and Signaling Molecule. Int. J. Mol. Sci..

[36] Miller V.J., Villamena F.A., Volek J.S. (2018). Nutritional Ketosis and Mitohormesis: Potential Implications for Mitochondrial Function and Human Health. J. Nutr. Metab..

[37] Wang X., Yu Q., Liao X., Fan M., Liu X., Liu Q., Wang M., Wu X., Huang C.-K., Tan R. (2023). Mitochondrial Dysfunction in Arrhythmia and Cardiac Hypertrophy. Rev. Cardiovasc. Med..

[38] Seefeldt J.M., Libai Y., Berg K., Jespersen N.R., Lassen T.R., Dalsgaard F.F., Ryhammer P., Pedersen M., Ilkjaer L.B., Hu M.A. (2024). Effects of Ketone Body 3-Hydroxybutyrate on Cardiac and Mitochondrial Function during Donation after Circulatory Death Heart Transplantation. Sci. Rep..

[39] Berg-Hansen K., Tinggaard A.B., Nielsen R., Jensen T.H., Gopalasingam N., Larsen A.H., Møller N., Böttcher M., Hollingdal M., Poulsen M.K. (2025). Should Exogenous Ketone Body Supplementation Be Considered in Patients Hospitalized for Acute Heart Failure? Rationale and Design of the KETO-AHF Trial. J. Card. Fail..

[40] Bear D.E., Ridley E.J., Daly K., De Waele E., Al Dabbous T., Karpasiti T., Stoppe C., Barrett N.A. (2026). 2025 ELSO Consensus Statement for the Provision and Management of Nutrition Therapy in Critically Ill Adult Patients Requiring Extracorporeal Membrane Oxygenation. ASAIO J..

[41] Monnet X., Messina A., Greco M., Bakker J., Aissaoui N., Cecconi M., Coppalini G., De Backer D., Edul V.K., Evans L. (2025). ESICM Guidelines on Circulatory Shock and Hemodynamic Monitoring 2025. Intensive Care Med..

